# Recognition–decision–execution: a new design principle for intelligent antimicrobial systems

**DOI:** 10.3389/fmicb.2026.1983088

**Published:** 2026-10-14

**Authors:** Jingwen Ma, Min Feng, Si Li

**Affiliations:** 1 Department of Clinical Laboratory Medicine, The First Affiliated Hospital of Dalian Medical University, Dalian, Liaoning Province, China; 2 The Institute of Pulmonology, The First Affiliated Hospital of Dalian Medical University, Dalian, Liaoning Province, China

**Keywords:** antimicrobial resistance, functional decoupling, mechanistic synergy, modular antibacterial systems, programmable antibacterial therapy, recognition–decision–execution

## Abstract

The continued rise of antimicrobial resistance highlights fundamental limitations of prevailing antibacterial strategies, which remain largely dominated by continuous, non-directed killing. Although combination therapies and multifunctional materials are frequently described as synergistic, many reported effects primarily reflect pharmacokinetic enhancement or local concentration increases, rather than genuine reprogramming of antibacterial behavior. As a result, most current approaches still impose sustained selective pressure and offer limited control over timing, location, and intensity of killing. Here, we propose the Recognition-Decision-Execution (RDE) framework as a function-driven organizational principle for antibacterial system design. In this framework, antibacterial activity is decomposed into three orthogonal yet logically coupled modules: pathogen recognition, decision-making, and killing execution. Recognition modules provide contextual information regarding pathogen identity, microenvironmental state, and infection dynamics; decision modules interpret these inputs to authorize action with defined spatiotemporal and intensity constraints; and execution modules eliminate pathogens only upon conditional approval. This architecture transforms antibacterial activity from a default, always-on process into a programmable, event-driven intervention that may reshape rather than universally reduce selective pressure. We discuss how functional decoupling enables a clear distinction between apparent (pseudo-) synergy and true mechanistic synergy, and how regulatory-execution interactions can qualitatively reshape killing mechanisms rather than merely amplifying them. Importantly, functional roles are not intrinsic to specific agents: antimicrobial peptides, nanomaterials, and nucleic acid aptamers can dynamically interchange between regulatory and execution functions depending on system architecture. The aptamer-nanomaterial-antimicrobial peptide triad serves as a minimal closed-loop example illustrating how sensing, decision-making, and execution can be independently optimized yet synergistically integrated. By emphasizing system logic, modular verifiability, and conditional authorization of killing, the RDE framework offers a unifying conceptual language for the design of programmable and adaptive antibacterial therapies. It should be emphasized that the RDE framework represents a unifying conceptual paradigm and set of testable design hypotheses. Most reported antibacterial examples only achieve partial pairwise functional slices rather than fully-validated ternary closed-loop RDE architectures.

## 1 Introduction

Traditional antibacterial strategies have largely conceptualized pathogens as independent and static entities that can be directly, rapidly, and potently suppressed or eliminated by a single molecular agent acting on a defined target (Mao et al., 2022). However, In traditional strategies, the use of a single drug or material often fails to effectively address complex pathogen infections due to the lack of a systematic, multi-dimensional approach (Husna et al., 2023). This limitation primarily arises from the inability of conventional strategies to integratively address multiple critical dimensions, including antibacterial efficacy, host protection, and resistance containment, as a consequence, these approaches frequently exhibit intrinsic shortcomings related to toxicity, stability, safety, and the precision of antimicrobial activity (Salam et al., 2023).

Although single-component materials, including nanomaterials, have demonstrated promise in catalytic antibacterial applications, their associated systemic risks remain difficult to disregard (Rasheed et al., 2024). Concerns regarding the long-term biosafety of nanomaterials, their potential immunogenicity, and the possibility of inadvertently driving adaptive responses in pathogens-such as enhanced biofilm formation-represent substantial barriers to their broader clinical translation (Jin et al., 2022; Wang et al., 2022; Wu et al., 2022). By contrast, aptamer-based technologies enable highly precise pathogen recognition (Li et al., 2023); however, they lack intrinsic bactericidal activity and therefore cannot independently fulfill the full spectrum of tasks required for the effective identification and elimination of pathogenic threats (DeRosa et al., 2023). These limitations collectively highlight a critical gap in current antibacterial strategies: against the backdrop of mounting global therapeutic challenges imposed by multidrug-resistant ESKAPE and other pathogens, an integrated framework capable of coordinating precise recognition, controllable decision-making, and effective bacterial elimination is still lacking (Miller and Arias, 2024).

In this review, we aim to systematically analyze emerging antibacterial strategies from a modular and system-level perspective, highlighting how functional decoupling and coordinated integration of recognition, decision, and execution can enable more precise, controllable, and evolution-aware antimicrobial interventions.

Despite substantial progress in multi-input responsive nanocarriers, logic-gated synthetic-biology circuits, pathogen-activated prodrugs, Trojan-horse antibiotics, stimuli-responsive nanomaterials, targeted delivery and closed-loop therapeutic platforms (Nazli et al., 2022; Deusenbery et al., 2021), most existing antimicrobial design paradigms focus on material or circuit engineering rather than establishing standardized, modular experimental benchmarks for discriminating pharmacokinetic potentiation from mechanistic synergy, nor formalizing omission-modulation validation workflows for condition-authorized antibacterial activation. This review elaborates the Recognition-Decision-Execution (RDE) concept, explicitly contrasts it against these established antimicrobial design strategies (Table 1), clarifies hypothetical versus experimentally verified claims, and proposes objective experimental benchmarks for modular antimicrobial systems. The core novelty of the RDE framework lies in three aspects: (1) functional decoupling of information-only recognition, multi-signal integrating decision-making, and conditional execution into independently omittable and verifiable modules, instead of direct signal-to-output coupling; (2) a suite of testable omission-modulation experimental benchmarks to separate pharmacokinetic potentiation from true mechanistic synergy; (3) shifting antibacterial design thinking from material-centric optimization toward system-level information-processing and evolution-aware intervention. We emphasize that individual components resembling RDE functions have been reported previously. Our contribution lies not in any single component, but in their integration into a unified organizational framework, together with experimentally testable principles for evaluating whether a system achieves coordinated recognition, decision, and execution.

**Table 1:** Comparison of representative antibacterial therapeutic paradigms.

System	Recognition module	Independent Decision-making module	Execution module	Programmability	Mechanistic synergy	Selective pressure characteristics	Experimental status
Conventional antibiotics	No dedicated context-sensing recognition layer	Absent; no separable decision/authorization layer	Antibiotic molecule	Limited at the system level	Not an inherent architectural feature; depends on drug combinations	Broad and exposure-dependent selection pressure	Extensive clinical use
AMP-nanomaterial delivery system	Targeting or localization; sensing is generally not a separable layer	Usually absent as a separable decision layer; stimulus–release coupling predominates	AMP and/or nanomaterial-mediated antimicrobial activity	Limited (tunable release kinetics)	Often reflects delivery/exposure enhancement; true mechanistic synergy requires independent validation	Continuous exposure	Numerous in-vitro studies; few clinical
Targeted nanocarrier	Usually absent as a separable layer; targeting is coupled to delivery/release	Absent; arrival triggers payload release	Various cargo	Limited	Primarily pharmacokinetic/exposure enhancement; mechanistic synergy requires independent validation	Continuous exposure	Pre-clinical
Stimuli-responsive antibacterial platform	One or more physicochemical or pathogen-associated stimuli	Stimulus–response coupling; independent logic/authorization layer is usually absent	Antibiotic/AMP/PTT	Moderate; stimulus sensitivity and response thresholds can be tuned	May generate functional interactions, but mechanistic synergy requires independent quantitative validation	Condition-dependent but no multi-logic gating	Pre-clinical
Phage system	High-specific host recognition	No readily separable programmable decision layer; replication and lysis are genetically coupled	Phage replication, progeny production and host lysis	Low to moderate; engineering is possible but constrained by phage biology	Not an inherent architectural feature; depends on phage–host or combination context	Strongly context-dependent; host-receptor loss and other escape mechanisms can be selected	Clinical phage therapy
Multi-input responsive nanocarrier	Two or more environmental or pathogen-associated cues	Multi-input response may be integrated within the material; independent authorization layer is not necessarily separable	Antibiotic/AMP/other cargos	Moderate to high; input combinations and response thresholds can be engineered	Can exhibit non-additive functional effects, but mechanistic attribution requires quantitative and orthogonal validation	Potentially discontinuous or context-dependent exposure; escape through loss of one input remains possible	Increasingly demonstrated in preclinical systems
Synthetic-biology logic circuit	Molecular, cellular, or pathogen-associated biological inputs	Explicit Boolean or analogous logic integration can conditionally regulate output	Genetic/biological therapeutic effector	High; inputs, logic operations, thresholds, and outputs can be engineered	Logic integration itself does not establish pharmacological or mechanistic synergy; requires independent functional validation	Potentially conditional and state-dependent; susceptible to pathway loss, circuit mutation, or phenotypic escape	Extensive experimental validation; mainly preclinical for antibacterial applications
Pathogen-activated prodrug	Pathogen-associated enzyme, metabolite, or physiological state	Activation is chemically coupled to the triggering signal; usually not an independently interrogable layer	Antimicrobial drug or active metabolite	Moderate; activation chemistry and payload can be modified	May enhance selectivity or potency; mechanistic synergy requires separation from activation/exposure effects	Conditionally activated exposure may reduce unnecessary off-target exposure, but does not inherently eliminate resistance selection	Multiple preclinical examples; some translational development
Trojan-horse antibiotic	Pathogen-specific uptake pathway, e.g., siderophore-mediated transport	Usually no independent decision layer; uptake determines intracellular access	Antibiotic payload	Limited to uptake ligand/linker and payload engineering	Primarily a targeting/delivery strategy rather than a mechanistic-synergy framework	Selection is shaped by uptake dependence and can favor alterations in uptake pathways	Established preclinical and clinical examples
RDE ternary closed-loop system	Non-lethal context-dependent state sensing	Present: multi-signal integration, logic-gate, adjustable threshold	Modular replaceable effector (AMP/antibiotic/PTT-PDT)	High: tune recognition, decision threshold, execution independently	True mechanistic synergy requires passing minimal binary validation tests	Discontinuous, environment-coupled; adaptive evolutionary burden shifted (hypothesis)	Very few complete ternary examples; mostly pairwise functional slices

## 2 Paradigm shift in antimicrobial strategies

The direct systemic application of single antimicrobial peptides (AMPs) or conventional antibiotics is frequently associated with substantial challenges. High local or systemic concentrations of individual agents increase the risk of cytotoxicity (Pirković et al., 2023), while the intrinsic coupling of decision-making and execution, where pathogen recognition is inseparable from killing, can result in collateral damage to the host’s commensal microbiota (Wang et al., 2024; Maier et al., 2024). In parallel, pathogens can rapidly acquire resistance through genetic mutations or the upregulation of efflux pump mechanisms, ultimately undermining therapeutic efficacy (Mwangi et al., 2019; Tawiah et al., 2025). Consistent with these observations, accumulating evidence indicates that single-target agents struggle to sustain long-term effectiveness within the inherently dynamic pathogen-host ecosystem (Paduszynska et al., 2020). Traditional antibacterial strategies mainly rely on single-factor, single-target action for direct bacterial killing, which inevitably imposes strong selective pressure as multidrug-resistant phenotypes continue to spread among ESKAPE pathogens (De Oliveira et al., 2020). Developing novel antibacterial strategies has therefore become a major research priority, yet conventional approaches fail to address bacterial infection dynamics, adaptive capacity and microenvironment-related features, breaking the complete antibacterial chain of “identify, kill, clear, repair” (Bjarnsholt et al., 2022). Its failure at the system level stems from the fact that the linear thinking of “single point breakthrough” cannot cope with the complex and changeable, non-linear changes of pathogens and microenvironments. These limitations have prompted the paradigm of antimicrobial therapy to shift from molecular-centric to “Dual synergy” and even “Antimicrobial systems engineering”.

By deliberately engineering multi-target engagement and multi-mechanistic coupling, synergistic antibacterial strategies can generate amplified therapeutic effects that extend beyond the simple additive action of individual components (Wang et al., 2025; Lei et al., 2025). Early forms of synergy, exemplified by combinations of antibiotic A and antibiotic B, largely remain additive in nature, primarily expanding antibacterial spectra, delaying resistance emergence, and enhancing bactericidal potency (Wojtyczka et al., 2014). However, such combinations rarely constitute true mechanistic synergy and typically exhibit limited efficacy against biofilms and multidrug-resistant pathogens (Hochvaldová et al., 2022).

In AMP-antibiotic regimens, AMPs frequently function as triggers that transiently weaken resistance barriers, promote antibiotic influx, and thereby enable substantial dose reduction (Ma et al., 2024; Chen et al., 2024; Ridyard et al., 2023). Combinations of AMPs with nanomaterials further extend synergy to structural and kinetic dimensions, facilitating targeted delivery and sequential or controlled release (Morton et al., 2013; Tang et al., 2021). By contrast, nanomaterial-antibiotic systems often rely on externally responsive cues, such as pH changes, light, or reactive oxygen species to enhance local drug accumulation, achieving material-driven activation while only partially compensating for intrinsic biological limitations (Manivasagan et al., 2024; Ioannou et al., 2023).

Although synergistic antimicrobial strategies have demonstrated clear advantages over monotherapy in terms of therapeutic efficacy and resistance management, most remain predominantly reliant on overlapping functional contributions to enhance antimicrobial performance. Notably, these approaches have not been conclusively shown to achieve durable resistance suppression, as they typically do not implement a structural separation of pathogen identification, decision-making, and execution. This conceptual limitation has, in turn, driven the emergence of multicomponent antimicrobial architectures that emphasize hierarchical organization, information responsiveness, and programmable cooperation through deliberate functional decoupling.

## 3 The need to decouple “recognition-decision-execution”

Most mainstream antibacterial strategies currently adopt a design logic in which target recognition, response decision-making, and killing execution are integrated within a single molecular or material entity. Such a “function-compressed” architecture can perform adequately against pathogens of relatively low complexity. However, in real-world infection contexts characterized by increased heterogeneity, dynamic phenotypic adaptation, and the ongoing evolution of drug resistance, this design paradigm increasingly reveals systemic vulnerabilities that are difficult to circumvent (Silver, 2011; Salazar et al., 2024). For conventional antibiotics, pathogen “recognition” is largely mediated by passive diffusion and chemical accessibility across bacterial membranes; membrane-permeation properties determine access to sites of action and directly trigger bactericidal activity (Sollima et al., 2024; Gamrat et al., 2025; Bermejo et al., 2004). In this context, recognition and execution are effectively inseparable, offering limited capacity to modulate the timing, magnitude, or spatial distribution of antimicrobial effects. AMPs largely operate under a similar paradigm: electrostatic interactions with negatively charged bacterial membrane components enable rapid recognition, which is immediately coupled to membrane disruption and bacterial killing (Dong et al., 2019). However, this contact-dependent recognition-execution mode similarly lacks an intermediate regulatory layer, leaving minimal scope for dynamic control. Collectively, when recognition, decision, and execution are compressed into a single functional entity, the resulting system functions primarily as a “blind executor” and cannot readily evolve into an intelligent antibacterial platform capable of addressing the complexity of real-world infection scenarios.

This highly coupled design paradigm not only constrains the scalability of antibacterial systems but also renders them largely dependent on a single mechanistic pathway, thereby creating pronounced vulnerability under evolutionary pressure (Fishovitz et al., 2014). When the critical route of action is circumvented or attenuated by the pathogen, the functionality of the entire system is compromised (Munford and Varley, 2006). Consequently, rather than continually layering additional functions within a single molecular or material entity, it may be more effective to deconstruct the fundamental operational units of the antibacterial process from a systems-engineering perspective (Figure 1).

**Figure 1:**
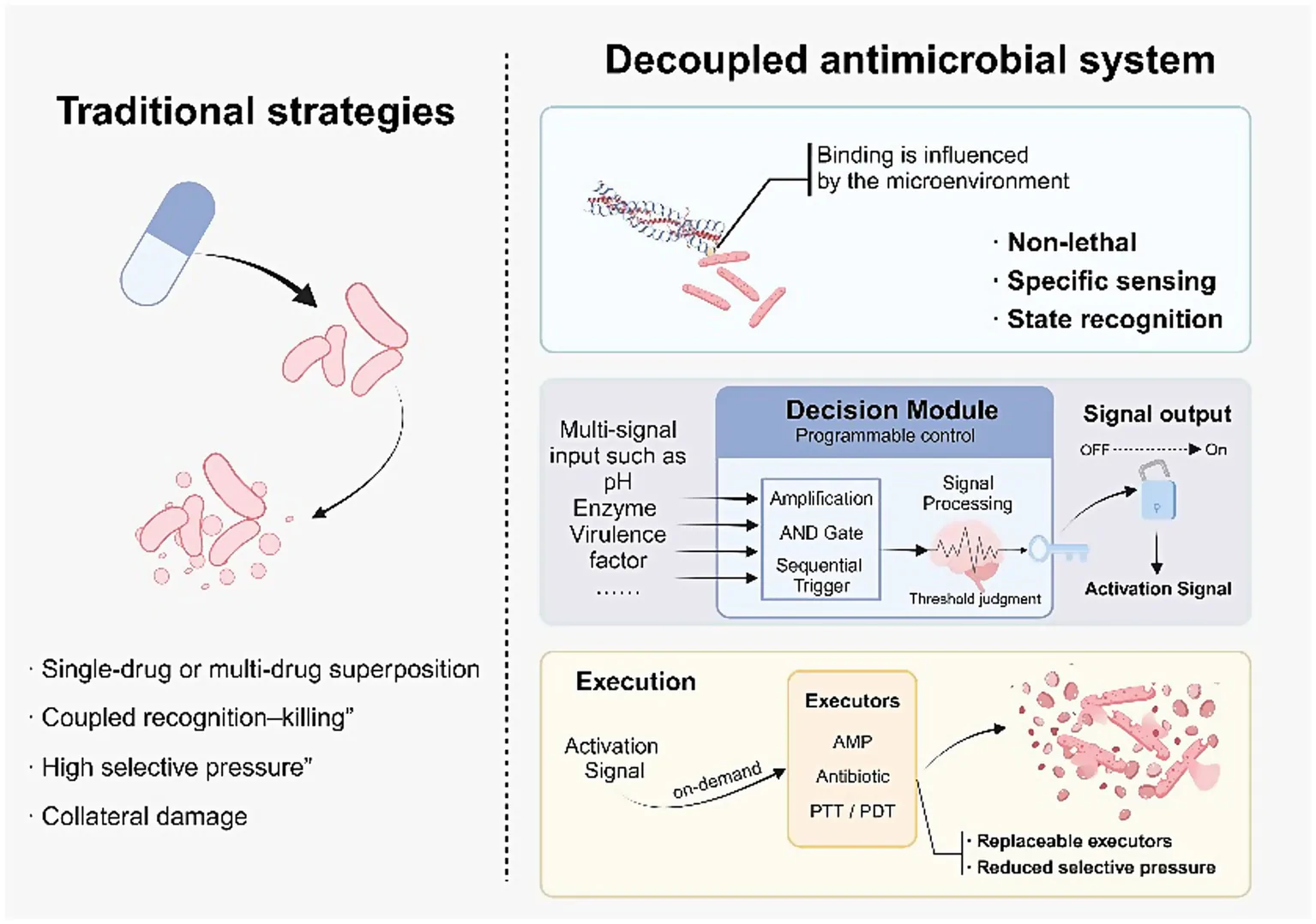
Functional decoupling transforms antibacterial killing from continuous pressure into an event-driven process. Conventional antibacterial therapies use single or combined drugs in which pathogen recognition and killing are inherently coupled, leading to continuous bactericidal activity, high selective pressure, and potential damage to host tissues and commensal microbiota. In a decoupled antimicrobial system, antibacterial activity is divided into three modules-Recognition, Decision, and Execution. Pathogens are first sensed through specific environmental or virulence signals. These inputs are processed by a programmable decision module, which activates the killing module only when predefined conditions are met. This on-demand strategy reshaping the temporal and spatial distribution of selective pressure and improves precision and safety.

Within this framework, it is conceptually significant to reconfigure the antibacterial process into three independents, hierarchically organized yet synergistically coupled functional modules: recognition, decision, and killing. Within this manuscript, “orthogonal” follows standard synthetic-biology meaning: biochemical and signal crosstalk between modules is minimized. Modular replaceability is a desired engineering outcome enabled by orthogonality, but it is not the definition of orthogonality itself. Despite low biochemical crosstalk, modules are logically coupled via transmitted information.

Notably, the decoupling of the regulatory layer from the execution layer mitigates the risk of reducing the system to mere “material-driven sterilization” and enables the incorporation of genuine programmability, verifiability, and strategic flexibility into the antimicrobial platform. Within this modular framework, the primary function of the recognition module is to accurately detect pathogens, the infected microenvironment, or relevant functional states, rather than to directly mediate killing. Suitable recognition units include nucleic acid aptamers lacking intrinsic bactericidal activity, phage-derived receptor-binding proteins, pathogen surface polysaccharide-binding elements, and molecular probes targeting virulence factors or specific phenotypic states. By decoupling specific recognition from toxic execution, it becomes possible to preserve recognition capacity during antimicrobial action and to reduce susceptibility to impairment under selective pressure. The decision module functions as the system’s central signal-processing unit, responsible for integrating, amplifying, and evaluating recognition signals, and translating them into programmable execution instructions.

A genuine decision module differs fundamentally from a simple stimulus-responsive trigger. A conventional trigger executes output immediately upon receiving one input signal. By contrast, a decision module integrates multiple independent input signals, implements logical operations (AND-gate, sequential trigger), and defines adjustable activation thresholds. An authorization signal is generated only when the composite signal exceeds threshold. Experimentally, decision-threshold validity can be probed by gradient-tuning individual input cues, single-input omission controls, and verifying that activation threshold can be shifted without altering recognition or execution components.

Platforms such as nanomaterials, stimuli-responsive polymers, and programmable self-assembled architectures provide an ideal basis for achieving sequential release, spatial confinement, and modulation of activity intensity. These features enable the design of nanostructures that shift the antibacterial process from passive, trigger-based responses toward condition-dependent, strategically executed interventions (Namakka et al., 2023; Zong et al., 2023; Xiao et al., 2024; Chen et al., 2024; Liu et al., 2024). Finally, as a replaceable execution unit, the killing module is responsible for pathogen elimination and does not require high target specificity. Broad-spectrum antimicrobial peptides, conventional antibiotics, or modalities such as photothermal and photodynamic effects can all serve as general-purpose execution tools. When operated under the precise guidance of the regulatory module, these effectors can achieve effective pathogen clearance while substantially mitigating continuous selective pressure on microbial populations (Zong et al., 2023; Xiao et al., 2024; Hu et al., 2022).

Overall, this engineering-oriented approach-centered on functional decoupling and modular synergy-transforms antibacterial agents from passive killing tools into multi-level systems endowed with sensing, decision-making, and execution capabilities. By integrating these hierarchical functions, such platforms offer a novel design paradigm for addressing complex infection scenarios and the persistent challenge of antimicrobial resistance.

While RDE builds upon prior advances in stimuli-responsive antimicrobials, targeted nanocarriers, theranostic agents and closed-loop therapeutic platforms, key conceptual differences can be defined as follows. Conventional stimuli-responsive systems follow a “single-signal-to-execution” logic: sensing input directly triggers payload release or activation without an independent intermediate decision layer integrating multi-input signals. Targeted delivery mainly improves pharmacokinetic biodistribution; killing activity remains constitutive once the effector reaches the target site, without conformational silencing gated by recognition criteria. Theranostic platforms combine diagnostic readout and therapy, but diagnostic signals are not used for logical authorization to suppress execution. Most reported closed-loop therapeutic systems rely on external device-based sensing rather than intrinsic molecular-level modular decoupling. In contrast, the core novelty of RDE lies in separating information-only recognition, programmable multi-signal decision-making, and conditional execution: recognition outputs sensing information without direct killing; the decision module performs multi-signal integration, logical gating and threshold evaluation to generate authorization; execution modules are kept silenced prior to authorization and can be independently removed for modular validation. This framework also delivers testable criteria for judging true mechanistic synergy, which has not been consistently formalized across these technology-specific paradigms (Table 1).

Related advances in synthetic biology and programmable therapeutics further illustrate the individual feasibility of RDE-like functions. Modular genetic logic gates have enabled bacterial systems to integrate multiple molecular inputs and generate defined outputs, providing examples of biological decision-making (Wang and Buck, 2014). Programmable bacterial receptors have also been developed to recognize clinically relevant biomarkers, demonstrating the feasibility of modular recognition systems (Chang et al., 2021). Similarly, engineered phages and programmable microbial therapeutics can couple target recognition with controlled antimicrobial or therapeutic outputs (Li et al., 2025). However, these approaches also reveal important limitations, including circuit complexity, narrow targeting ranges, evolutionary escape, biocontainment, and translational barriers (Oromí-Bosch et al., 2023). Thus, RDE should be viewed as a conceptual framework that integrates and organizes these previously developed functional principles rather than as a claim that recognition, decision-making, or execution mechanisms are individually novel.

## 4 Ternary modular synergy: reprogramming antibacterial logic through recognition-decision-execution decoupling

Functional decoupling alone does not define a viable antibacterial system; rather, it establishes structural constraints on how antimicrobial functions can be organized. Within this framework, any effective platform must accommodate at least three orthogonal functions: pathogen recognition, response modulation, and killing execution. These modules collectively form a closed functional loop: recognition provides information input, decision converts information into decision signals, and execution achieves physical clearance. Fully integrated ternary antimicrobial systems remain uncommon, largely due to experimental limitations. Consequently, most reported studies achieve only partial decoupling, typically manifested as pairwise module cooperation. Such pairwise interactions should not be interpreted as independent strategies but rather as functional slices of a decoupled antibacterial system. Importantly, it remains necessary to distinguish true mechanistic synergy from apparent synergy arising from pharmacokinetic enhancement, with consideration of specific application contexts and functional requirements.

### 4.1 Decision-execution

In analyses of functional slices involving the regulatory and execution modules, it is critical to distinguish apparent, pseudo-synergistic effects-arising from improved pharmacokinetics or simple increases in drug concentration-from genuine synergistic interactions occurring at the mechanistic level. The former typically manifests as a quantitative enhancement of bactericidal activity, whereas the latter entails qualitative changes in antimicrobial pathways, temporal coordination, or functional roles. Importantly, the system-level implications of these two types of synergy are fundamentally distinct.

In current antibacterial design practices, AMPs and nanomaterials are often treated as prototypical representatives of the killing execution and regulatory modules, respectively. However, this functional assignment is not intrinsic to the agents themselves, but rather reflects the inertia of traditional classification schemes. In fact, depending on the application context and system architecture, AMPs and nanomaterials can dynamically interchange between regulatory and execution roles. Such role reassignments consequently redefine the underlying coordination mechanisms within the antimicrobial system. In many early studies, Nanomaterials primarily served as delivery and protective platforms, reducing the risk of AMP degradation by proteases through physical encapsulation or surface modification, while enabling sustained release and local enrichment to enhance overall antibacterial efficacy (Xiao et al., 2024). For example, Almaaytah et al. encapsulated AMPs within nanomaterials to protect them from rapid degradation and to enable gradual release in the vicinity of bacterial cells, thereby increasing local drug concentrations. Experimental results demonstrated that the AMP-Nano complexes exhibited stronger antibacterial activity than free AMPs; however, this enhancement was largely attributable to prolonged AMP action and reduced biotoxicity, in addition to the intrinsic bactericidal mechanism of the AMP itself. Importantly, the nanomaterials did not introduce new antimicrobial pathways, but rather optimized delivery efficiency and molecular stability, consistent with pseudo-synergy arising from pharmacokinetic improvements (Almaaytah et al., 2017).

Similarly, in the study by Casciaro et al., the role of nanomaterials was to protect AMPs, facilitate transport to the infected lung tissue, and sustain local concentrations via slow release. While this approach addresses the challenges of peptide degradation and limited penetration through mucus or biofilm, it does not alter the intrinsic mechanisms of either the AMP or the nanomaterial itself (Casciaro et al., 2019). A subset of research has moved beyond pharmacokinetic enhancement of single-drug combinations to achieve mechanistic-level coupling between the regulatory and execution modules. For instance, Casciaro et al. demonstrated that the incorporation of nanomaterials substantially altered the mode of action of AMPs. Unlike free AMPs, which rely on stochastic single-molecule interactions with bacterial membranes, AMP-Nano complexes form high-density aggregates on the bacterial surface, markedly accelerating membrane disintegration. Electron microscopy confirmed that the complex induced collapse of bacterial cell structures, rather than solely relying on the intrinsic antimicrobial mechanism of the peptide. Through coordinated Nano-mediated decision and AMP-driven killing, the system achieves an enhanced linkage of charge targeting and membrane perturbation, effectively establishing a novel bactericidal mechanism (Casciaro et al., 2017).

This observation illustrates that when nanomaterials influence not only the spatial localization of AMPs (“where AMPs go”) but also their mechanistic action (“how AMPs act”), synergy transitions from the pharmacokinetic to the mechanistic level. Further studies have highlighted that the roles of regulatory and execution modules are not fixed and can be dynamically reconfigured. In the work by Ruden et al., AMPs functioned as regulatory gates, modulating Nano activity to facilitate intracellular entry, which in turn amplified AMP-mediated membrane disruption. This interaction generates a true mechanistic synergy, consistent with a “1 + 1 > 2” effect (Ruden et al., 2009). In this system, as shown in Figure 2, AMPs and nanomaterials assume switchable regulatory and execution roles, exemplifying the strategic flexibility afforded by modular decoupling and functional reconfiguration.

**Figure 2:**
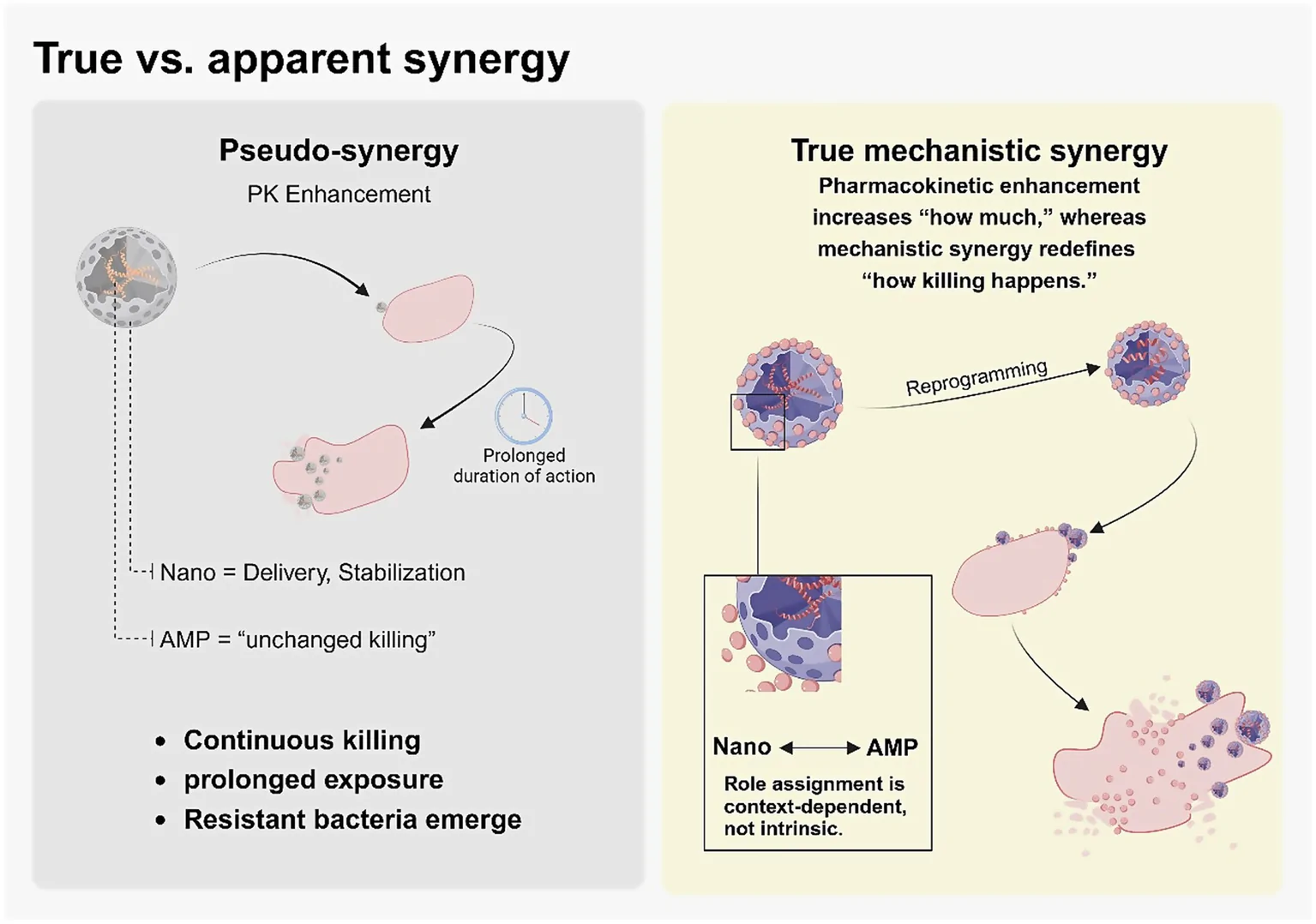
Distinguishing apparent synergy from true mechanistic synergy in antibacterial combinations. Apparent (pseudo-) synergy arises primarily from pharmacokinetic enhancement, such as improved delivery, stabilization, or prolonged local exposure. In these systems, nanomaterials mainly function as carriers, whereas the intrinsic killing mechanism of AMPs remains unchanged. The resulting antibacterial effect reflects quantitative amplification (“how much” killing), leading to continuous exposure and sustained selective pressure that promotes resistance emergence. True mechanistic synergy emerges when functional roles are reprogrammed at the system level. Here, nanomaterials and AMPs interact in a context-dependent manner, reshaping spatial organization, interaction density, or execution pathways, thereby redefining “how killing happens”. Such mechanistic coupling generates qualitative changes in antibacterial action and demonstrates that regulatory and execution roles are not intrinsic to specific agents but dynamically assigned by system architecture.

Overall, pre-assigned or fixed roles within antimicrobial pathways can promote the emergence of resistance under strong selective pressure (Kuijk et al., 2023). Functional decoupling of regulatory and execution modules, by avoiding rigid role assignments, enables the development of novel antimicrobial agents and opens a new design space for constructing programmable and adaptive antibacterial strategies.

### 4.2 Recognition + execution

Within the framework of the recognition-killing functional slice, the role of the recognition module should not be reduced to a mere “targeting tag” or simple localization tool. The critical function of effective recognition lies not in answering “is it bacteria?” but in determining to whom, when, where, and with what intensity the response should be directed. Recognition provides sensory input signals; authorization itself originates exclusively from the decision module integrating these inputs. Only when sensory information from recognition feeds into the decision module and satisfies predefined logical thresholds does killing transition from blind execution to precisely-triggered event. This hierarchical recognition and control directly reduces the exposure of the execution module to healthy tissues while enhancing bactericidal efficiency. As a result, pathogens are no longer continuously subjected to indiscriminate killing pressure, and the available evolutionary space for resistance is correspondingly constrained.

Building on this design concept, AMPs have been combined with high-affinity, high-specificity nucleic acid aptamers and supplemented by nanodelivery platforms to develop a representative class of “recognition-gated” antibacterial strategies. Unlike conventional targeted delivery, the central principle of these systems is not merely to increase AMP accumulation at the infection site; rather, it is to enhance the precision of AMP targeting, such that the execution of the killing process itself is constrained and regulated by programmable molecular recognition (Li et al., 2023; Drayton et al., 2020). The study by Lee et al. provides a clear experimental demonstration of this concept. In the constructed system, DNA aptamers mediate the specific recognition of target bacteria, gold nanoparticles serve as structural scaffolds and delivery platforms, and AMPs execute membrane-disruptive killing. While the fundamental bactericidal pathway of the AMP remains unchanged, the results indicate that aptamer incorporation significantly alters the conformational state of AMP and its nanoscale spatial arrangement. These conformational and spatial changes render AMP activation no longer spontaneous but precisely regulated. Consequently, AMP exhibits enhanced intracellular penetration, more directed retention at the bacterial membrane, and finely tuned activation, collectively resulting in substantially improved bactericidal efficacy (Lee et al., 2017). In this system, the role of aptamers extends beyond that of a conventional “passive targeting tag.” They function as programmable molecular regulatory elements: on one hand, they mediate the specific binding of AMPs; on the other, their intrinsic structural properties impose constraints on the timing of AMP activation, the location of release, and the spatial scope of action. As a result, killing execution is no longer a default event, but a process that is “authorized” to proceed only when defined recognition criteria are satisfied.

The concept of “recognition as a gating mechanism” is further exemplified in the study by Liu et al. In this work, a pH-responsive nanocarrier system was constructed, whose drug release is regulated not by a single factor but by the combined influence of local pH changes in the infected microenvironment and the activity of bacterial secretases. These carriers remain highly stable in the absence of bacterial lipases or when specific acidic thresholds are not reached. Compared with conventional soluble drugs or nanosystems that rely solely on physical encapsulation for delivery, this dual-regulated system effectively prevents premature drug leakage in the bloodstream or non-infected tissues. Only upon reaching the specific infection microenvironment-such as within biofilms-does the carrier respond, releasing the antibacterial payload and thereby imposing well-defined spatial and temporal boundaries on the killing process (Liu et al., 2018).

Compared with single-threshold or unconditional release systems, dual- or multi-conditional response strategies more fully illustrate how recognition-derived information can be converted into conditional authorization through a decision layer. Within such a modular antibacterial framework, recognition is no longer a one-time localization event but a continuous regulatory process that dynamically constrains and precisely authorizes execution. In other words, the recognition module should not merely answer the question, “Where do antimicrobial components accumulate?” but also determine whether, when, and in what manner the bactericidal mechanism is activated. Although direct experimental evidence demonstrating synergy between metabolic sensors, enzyme-specific recognition, or virulence factor sensing and AMP activity remains limited, current studies provide clear design principles and feasible approaches. These insights lay a foundation for the development of higher-order, recognition-gated antibacterial systems with enhanced programmability and intelligent functionality.

### 4.3 Recognition + decision

In most existing antibacterial strategies, whether employing conventional antibiotics or AMPs, the killing execution module generally lacks inherent directionality. Once activated, killing is typically non-selective, exerting sustained pressure not only on the target pathogen but also, inevitably, on healthy cells and tissues (Yao et al., 2023; Stojowska-Swędrzyńska et al., 2023). By contrast, bacteriophage-based antibacterial systems have long been recognized as “potential functional units” due to their intrinsic high specificity for target bacteria and their self-regulatory replication-amplification properties. Fundamentally, bacteriophages embody a killing execution system that integrates its own recognition and regulatory logic, offering valuable insights for the design of next-generation antibacterial platforms (Peng et al., 2025).

If killing execution units such as AMPs are conceptualized as antimicrobial effectors, it is not the effectors themselves that determine whether, when, and how they act, but the upstream recognition and regulatory modules. By integrating a recognition unit with precise target selection and temporal control into an AMP, and coupling it with a highly flexible regulatory module, the system can be programmed to “release how much, for how long, and with what intensity.” In essence, this approach transforms the recognition-decision slice into an editable guidance system for space, time, and intensity. Within this framework, the killing module ceases to act as a source of continuous selective pressure and instead functions as an execution tool under precise guidance. This design both accomplishes the bactericidal task and substantially reduces the evolutionary pressure for resistance development.

Existing studies have demonstrated multiple feasible pathways for recognition and guided antibacterial action. For example, Thorn et al. utilized bacteria-specific enzymes as recognition signals to trigger antibiotic release from liquid crystal nanoparticles that are digestible by bacterial lipases. This design initiates killing execution exclusively in the presence of the pathogen, markedly improving the treatment of multidrug-resistant bacteria (Thorn et al., 2020). In this system, recognition transcends mere localization and directly determines whether the killing module is authorized to act. Similarly, nanocarriers engineered for specific recognition via surface modifications have been employed to confine killing activity to infection-associated cells. For instance, macrophage membrane-encapsulated nanosystems can mimic host cell properties, actively home to sites of infection, and release the antibacterial agent epigallocatechin-3-gallate (EGCG) in the presence of bacteria. This approach has been applied to treat bacterial infections, effectively eliminating intracellular pathogens while limiting off-target effects (Tang et al., 2024).

In addition to molecular recognition, the infectious microenvironment itself provides critical regulatory cues. Sites of bacterial infection are typically characterized by local acidity, elevated temperature, and increased oxidative stress. Accordingly, smart antibacterial materials can be engineered to undergo conformational changes or release drugs in response to specific pH conditions. Temperature fluctuations may also serve as triggering signals, and the local abundance of reducing molecules such as glutathione (GSH) can be harnessed to regulate redox-responsive release systems (Manivasagan et al., 2024; Zhang et al., 2024). At the level of the regulatory module, the responsiveness of smart materials to endogenous or exogenous stimuli further enhances the degree of guidance over antibacterial action. Signals such as light, magnetic fields, ultrasound, temperature, pH, enzyme activity, and redox potential can be harnessed to precisely control the timing, location, and rate of drug release (Zhang et al., 2024). For example, light-triggered lysis of red blood cell carriers has been demonstrated to enable highly spatiotemporally precise delivery of protein therapeutics (Darrah and Deiters, 2021), while 3D printing technologies allow the fabrication of drug formulations with customized release profiles (Deng et al., 2025). Furthermore, multilayer structural designs-such as a core-drug layer-protective coating-controlled-release polymer configuration-provide a structural basis for long-term sustained release and staged, multi-phase drug delivery (Trofimiuk et al., 2019). In contrast, certain nanomaterial-based antibacterial strategies that rely on physical disruption mechanisms-such as photothermal or sonodynamic effects-or on multi-modal synergistic actions exhibit bactericidal activity that is largely independent of antimicrobial resistance (AMR), and in some cases even suppress the emergence of new resistance phenotypes (Xie et al., 2023) (Figure 3).

**Figure 3:**
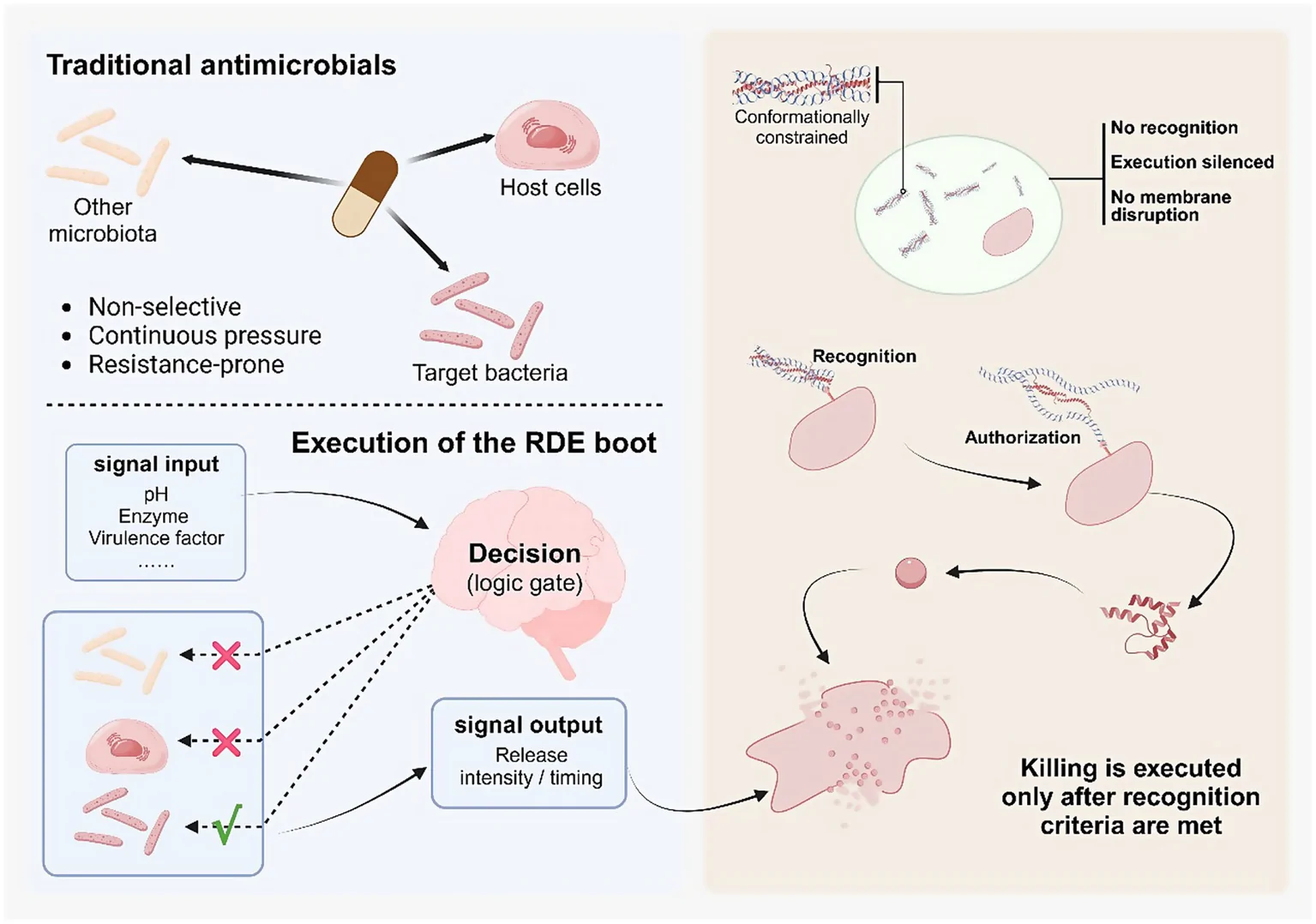
Event-driven antibacterial execution enabled by recognition-decision coupling in the RDE system. Traditional antimicrobial agents act in a non-selective manner, simultaneously affecting target bacteria, commensal microbiota, and host cells, thereby imposing continuous selective pressure and increasing resistance risk. In the RDE system, multi-dimensional signals from the infection microenvironment-such as pH, enzyme activity, or virulence factors-are integrated by a programmable decision module implementing logical gating and threshold evaluation. Only signals that satisfy predefined criteria generate an activation output. Recognition constrains execution by maintaining killing modules in a silenced or conformationally restricted state in the absence of authorization. Upon successful recognition and decision approval, execution is precisely activated, enabling targeted and conditional bacterial killing. This architecture converts antimicrobial action from continuous pressure into a mandated, event-driven process, enhancing precision while reducing collateral damage and evolutionary pressure for resistance.

Consequently, the integration of well-designed recognition-decision modules with long-acting delivery systems offers the potential for drug release over weeks, months, or even longer, thereby markedly improving patient compliance and reducing dosing frequency (Li et al., 2022). For instance, incorporation of tannic acid-iron complexes into chitosan films has been shown to substantially prolong the local antibacterial activity of gentamicin, illustrating how the regulatory module can finely orchestrate the timing of killing execution (Chevallier et al., 2023). In this context, the recognition-decision module represents the functional slice with the highest degree of engineering flexibility and the greatest plasticity within contemporary antibacterial systems. Its value lies not in substituting for lethal execution, but in precisely directing it, transforming killing into a “mandated event” rather than a process of continuous selective pressure.

### 4.4 From apparent enhancement to true synergy: system-level criteria for modular antibacterial integration

Within the “recognition-decision-execution” framework, the combination of any two modules should not be casually labeled as “synergy” solely because the antibacterial effect is enhanced. The critical question is whether the interaction induces deeper mechanistic changes-whether functional roles have been reassigned, the activation logic reshaped, or at least a qualitative alteration in the antimicrobial pathway of one component. Numerous studies indicate that mere juxtaposition of different functional units often results only in a linear increase in bactericidal efficiency without affecting the operational logic of the antibacterial system itself. Superficially, such effects may be described as “enhancement,” but at the system level, they represent a simple superposition of functions rather than true synergy: efficiency is improved, but logic remains unchanged. Within the RDE framework, mechanistic synergy is interpreted as requiring both quantitative evidence of non-additive interaction and independent evidence that the interaction alters the operational mechanism of antibacterial action. Hence, synergy is not purely a quantitative phenomenon, but a structural one, emerging only when functional decoupling is maintained and information can be reliably transmitted, amplified, and interpreted between modules.

When the recognition module is employed not merely for localization but to provide information on whether, when, and where to initiate action, and the regulatory module is capable of interpreting this information to generate precise spatiotemporal and intensity decisions, the activation of the killing module becomes conditionally authorized rather than default. Under these circumstances, antibacterial activity transitions from a state of continuous pressure to an event-driven process. This shift compresses the window for resistance selection while simultaneously enhancing system-level efficacy and safety.

From this perspective, the cooperation of paired modules should not be regarded as independent antibacterial strategies. Rather, they constitute a functional slice of a decoupled antibacterial system-a directional, localized structure extracted from the overall system. The value of such slices lies not in their “strength” per se, but in whether they establish information flow across modules. True synergy emerges only when this enhancement is accompanied by a reconfiguration of action pathways, a shift in triggering logic, or a reassignment of functional roles, representing a qualitative transition from pharmacokinetics-driven “pseudo-synergy” to mechanism-level “true synergy” (Baeder et al., 2016).

### 4.5 Experimentally testable criteria for true mechanistic synergy

Conventional quantitative frameworks for evaluating combinatorial antibacterial activity include Bliss independence, Loewe additivity, fractional inhibitory concentration index (FICI, checkerboard assay), and time-kill kinetics (Baeder et al., 2016). Under these classical definitions, improvements originating purely from pharmacokinetic effects (elevated local concentration, prolonged half-life, improved delivery) do not qualify as true synergy. Within the RDE framework, we further distinguish three categories of combinatorial outcome: (1) potentiation: sub-threshold effector gains activity via partner components without intrinsic mechanistic reprogramming; (2) pseudo-synergy: apparent enhanced efficacy driven solely by pharmacokinetic improvement, which fails to meet quantitative synergy thresholds from Bliss/FICI assays; (3) true mechanistic synergy: satisfying classical quantitative synergy thresholds together with evidence of qualitative reprogramming of antibacterial pathways via modular coupling.

To assign an interaction as true mechanistic synergy under the RDE framework, all of the following experimental benchmarks must be fulfilled:

1 Classical quantitative criteria for combinatorial antibacterial activity include the widely-adopted empirical threshold FICI ≤0.5 for checkerboard assays (derived from the Loewe additivity model) OR a statistically-significant positive synergy score relative to Bliss-independence expectations derived from time-kill measurements. Note that FICI≤0.5 represents a community-accepted empirical convention rather than a regulatory mandatory breakpoint; no universal fixed magnitude cutoff exists for Bliss-derived synergy scores, and statistical significance against the null expectation of zero interaction is required (Baeder et al., 2016).
2 Modulate-removal/omission controls are performed: removing the recognition module substantially suppresses bactericidal activity beyond simple reduction in local compound accumulation; removing the decision-gating module abolishes conditional restriction of killing output.
3 Independent experimental evidence demonstrates qualitative reprogramming of killing pathways (e.g., conformational alteration of effector molecules, changed membrane disruption mode, modified spatial assembly on bacterial surface), rather than only elevated local concentration or prolonged half-life.

Role reassignment between regulatory and execution components (original criterion 4) represents supportive mechanistic evidence but is not treated as a mandatory experimental readout for true mechanistic synergy. If combinatorial antibacterial enhancement is observed without satisfying benchmarks 1–3, the effect should be categorized as potentiation or pseudo-synergy (Table 2).

**Table 2:** Summary of reported antibacterial platforms relevant to RDE-derived design concepts.

Recognition mechanism	Decision strategy	Execution effector	Target pathogen (as described in text)	Implementation level	Key limitations (as implied by text)	Ref.
No pathogen-specific recognition; nano-mediated delivery only	None; sustained release/local enrichment only	Antimicrobial peptide (AMP) encapsulated in nanomaterial	Not specified in text	Delivery-only pseudo-synergy (recognition–execution slice, no decision layer)	Enhancement limited to prolonged AMP action and reduced biotoxicity; no new killing mechanism introduced	Almaaytah et al. (2017)
Mucus/lung-tissue delivery; no specific bacterial recognition	Slow-release kinetics only	AMP delivered by nanomaterial to infected lung	Pulmonary infection (as discussed)	Delivery-only pseudo-synergy (recognition–execution slice, no decision layer)	Addresses peptide degradation and biofilm/mucus penetration, but does not alter intrinsic AMP or nanomaterial mechanism	Casciaro et al. (2019)
Charge-based bacterial surface interaction; no independent state-sensing	Nano-mediated spatial reorganization and aggregate formation on bacterial surface	AMP + nanomaterial composite; accelerated membrane disintegration	Not specified in text	Mechanistic-level decision–execution slice (true mechanistic synergy)	Nanomaterial influences “how AMPs act” rather than only “where AMPs go”; recognition module remains weak	Casciaro et al. (2017)
Not explicitly described as a dedicated recognition step	AMPs function as regulatory gates modulating nano-mediated intracellular entry	Reciprocal roles: AMP regulates nano activity; nano amplifies AMP membrane disruption	Not specified in text	Reciprocal decision–execution slice (role reassignment demonstrated)	“1 + 1 > 2” effect shown, but no independent multi-signal decision layer	Ruden et al. (2009)
DNA aptamer specific recognition of target bacteria	Aptamer imposes conformational/spatial constraint on AMP activation; gating of timing and release	Gold nanoparticle scaffold + AMP (membrane disruption)	As discussed (aptamer-targeted bacterium)	Recognition–execution slice with gating function	AMP bactericidal pathway itself unchanged; no independent programmable multi-signal decision module	Lee et al. (2017)
Dual microenvironmental sensing: local acidic pH + bacterial secretase/lipase activity	Dual-condition gating: carrier remains stable unless both pH threshold and lipase activity are met; prevents premature leakage	Antibacterial payload (antibiotic) released from pH−/enzyme-responsive nanocarrier	Biofilm-forming bacteria (as discussed)	Recognition–decision slice (multi-condition logical gating)	Execution effector cannot be independently swapped; carrier design is effector-specific	Liu et al. (2018)
Bacterial lipase as recognition trigger	Lipase-dependent digestion of liquid-crystal nanoparticle to initiate release	Antibiotic released from enzyme-degradable liquid-crystal nanoparticles	Multidrug-resistant bacteria (as discussed)	Recognition–decision slice (enzyme-triggered authorization)	Recognition relies on a single enzyme signal; lacks non-lethal state-sensing and multi-input logic	Thorn et al. (2020)
Macrophage-membrane coating; host-cell mimicry and active homing to infection sites	Bacteria-triggered release of encapsulated cargo	Epigallocatechin-3-gallate (EGCG) from macrophage-membrane-encapsulated nanosystem	Intracellular pathogens (as discussed)	Recognition–decision slice (biomimetic targeting + triggered release)	Specific molecular recognition logic remains limited; relies on host-cell mimicry rather than programmable decision threshold	Tang et al. (2024)
Single microenvironmental cue sensing (pH, temperature, GSH redox state)	Single-condition stimulus-triggered cargo release	Antibiotics, AMP and other payloads	Various infection-associated bacteria	Recognition-decision slice (single-condition response)	Only single-input signal supported, no multi-signal logical operation; risk of nonspecific premature payload leakage	Manivasagan et al. (2024); Stojowska-Swędrzyńska et al. (2023); Zhang et al. (2024)
Exogenous field signal sensing (light, magnetic field, ultrasound)	External-field-triggered physical killing or payload release	PTT/SDT physical modalities, antibiotics	Drug-resistant bacteria, biofilm-associated bacteria	Recognition-decision slice (externally controlled triggering)	Requires external equipment stimulation; lacks non-lethal sensing of pathogen intrinsic state	(Peng et al. (2025); Zhang et al. (2024); Darrah and Deiters (2021); Deng et al. (2025)

## 5 A conceptual framework for ternary modular assembly in recognition-decision-execution systems

We propose a ternary modular antibacterial system (named as RDE system) derived from traditional composite antibacterial strategies, envisioned as a programmable conceptual framework rather than a fully realized therapeutic platform.

### 5.1 Why ternary modular systems are not simple additions

The RDE system represents a function-driven organizational principle that deconstructs the antibacterial process into three orthogonal functional modules: pathogen Recognition, response Decision, and Execution. Through information transfer and logical coupling, these modules operate collaboratively to achieve system-level antibacterial function. Within this framework, the recognition module senses pathogens, the infectious microenvironment, or their functional states and provides critical informational input, while the killing module executes physical or chemical clearance only upon authorization. Together, these modules form a closed functional loop that transforms antibacterial behavior from passive, continuous killing into a conditionally triggered, programmable, and verifiable event-driven process. As shown in Figure 4, the nucleic acid aptamer-nanomaterial-antimicrobial peptide triad exemplifies a microcosm of this ternary closed loop, in which sensing, decision-making, and execution are independently optimized yet synergistically integrated.

**Figure 4:**
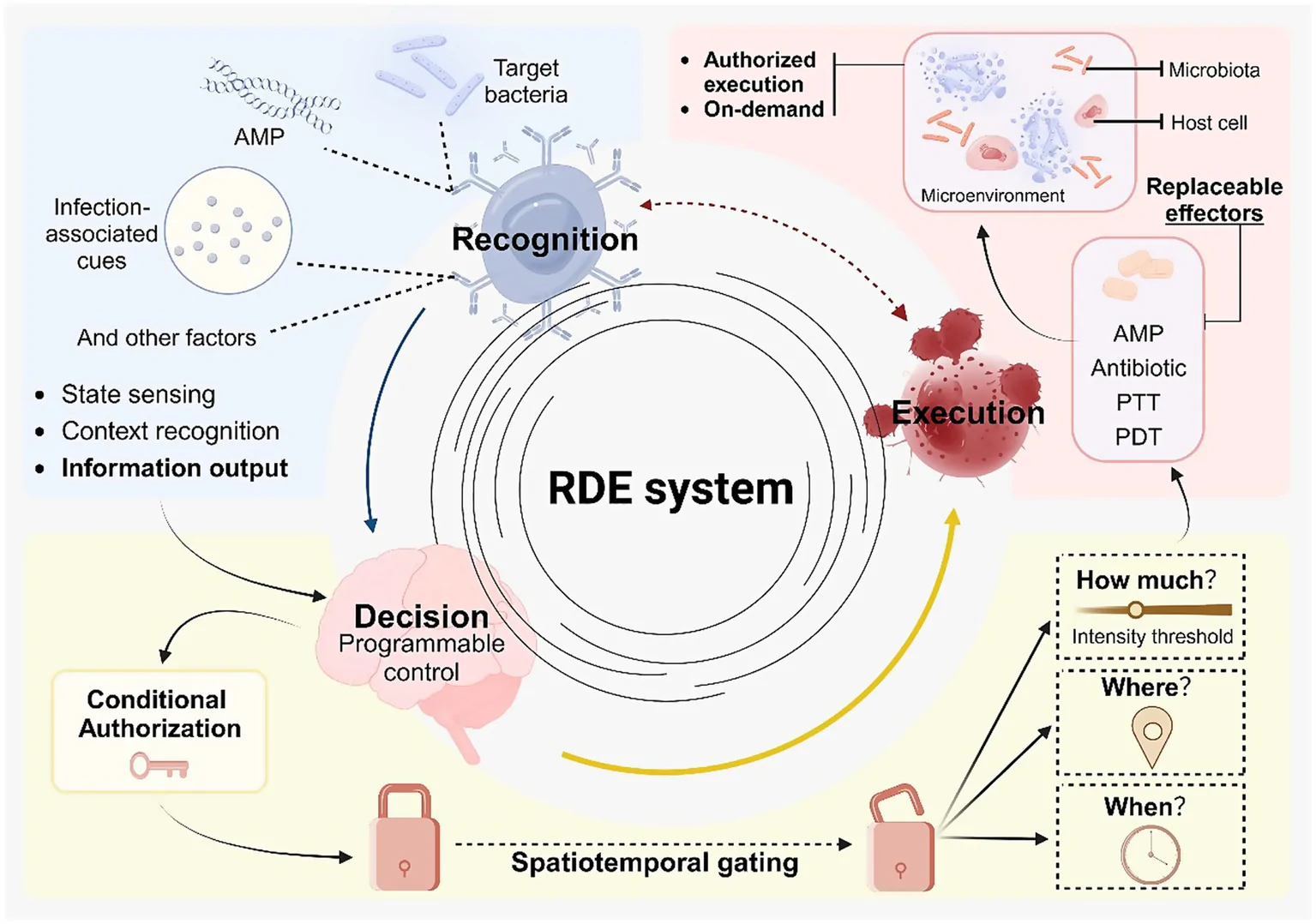
Conceptual architecture of the RDE framework as a closed-loop, conditionally authorized antibacterial system. The RDE framework is conceptually organized into three orthogonal yet logically coupled functional modules: recognition, decision, and execution. The recognition module performs non-lethal, context-dependent sensing of target bacteria and infection-associated cues, providing state sensing and information output. These signals are conceptually transmitted to a programmable decision module, which integrates multidimensional inputs to enable conditional authorization through logical gating and threshold evaluation. Upon authorization, the execution module activates replaceable effectors, including antimicrobial peptides, antibiotics, and physical modalities such as photothermal or photodynamic therapy, in an on-demand manner. The decision module is proposed to determine *how much, where,* and *when* execution occurs through spatiotemporal gating and intensity control. Importantly, this figure represents a conceptual RDE architecture rather than a fully experimentally validated ternary RDE system. The illustrated modules and their coupling are derived from experimentally demonstrated components and mechanisms reported in the literature, whereas their integration into a complete RDE architecture remains to be experimentally validated. Thus, the framework is presented as a conceptual and testable design paradigm rather than an established therapeutic architecture.

### 5.2 Functional decoupling of recognition, decision and execution

The synergy of the ternary system does not arise merely from an increased number of components, but from a fundamental reconfiguration of antibacterial behavior, underpinned by three key principles:

First, decision-making is decoupled from execution. In traditional antibacterial strategies, recognition is inherently conflated with killing, and contact is directly equated with execution; the system rarely incorporates a distinct “whether to kill or not” decision-making step. In the RDE system, by contrast, killing is authorized only by the regulatory module, rendering execution no longer a passive event but the outcome of a conditionally triggered decision. Second, spatiotemporal decision. The regulatory module confines execution to a defined location, temporal window, and intensity, transforming the antimicrobial process from continuous exposure into conditionally triggered action. This targeted control reduces nonspecific damage to healthy tissues and may compress the time window for resistance-related selection (hypothesis requiring further experimental validation). RDE architecture does not necessarily reduce overall magnitude of selective pressure; instead it reshapes selection into discontinuous, environment-coupled patterns and may give rise to alternative bacterial escape pathways, while simultaneously improving therapeutic safety and efficacy. Finally, modular disassembly and verifiability. Each module within the RDE system can be independently removed or replaced, allowing the contribution of individual components to overall system performance to be rigorously evaluated. This approach transforms the system from an empirical accumulation into a mechanistically grounded framework, where hypotheses can be systematically tested and falsified at each functional layer.

### 5.3 Design principles of ternary antimicrobial systems

To prevent the RDE system from degenerating into a mere multi-component superposition, its design should adhere to several guiding principles:

i Identification must precede execution. Sensing and recognition modules, such as aptamers, should be fully engaged before the release or activation of killing modules, such as antimicrobial peptides, to ensure that lethal action is constrained by the recognition signal rather than operating as a “one-size-fits-all” response.
ii The incorporation of the regulatory module does not perturb the conformational dynamics of the recognition module. Regulatory elements, such as nanomaterials, introduced into the system do not compromise the gating function of the recognition module.
iii The dosage of the lethal execution module should remain below the threshold required for standalone activity. Synergistic effects can be attributed to mechanistic-level amplification, rather than mere pharmacodynamic enhancement, only when substantial bactericidal efficacy is achieved at these sub-threshold doses.

We note that sustained sub-inhibitory exposure is a well-documented driver of antimicrobial resistance. Within RDE systems, sub-threshold dosing is only acceptable when killing activation remains strictly event-triggered and discontinuous, to avoid chronic low-level selective pressure. Continuous sub-threshold exposure should be avoided.

i Each module within the system can be independently disassembled and experimentally validated. Removal of any single module should lead to a predictable and directional reduction in antimicrobial efficacy; failure to observe such an effect would indicate insufficient evidence to support the proposed decoupling of system functions.
ii The directionality of the collaborative pathway should be clearly defined, following the sequence of Recognition-Decision-Execution rather than a random arrangement. This ensures that killing is precisely guided and prevents erroneous or excessive antimicrobial activity arising from disordered recognition or regulatory mechanisms.

In the RDE system, the synergy of the ternary modules is not defined by “what to do more”, but by “what to do under specific conditions”.

## 6 From concept to feasible implementation

### 6.1 Design of binary minimal verification units

In multi-component antibacterial systems, experimental outcomes are often confounded by multiple variables, such that apparent “true synergy” may be reduced to “pseudo-synergy”, reflecting local concentration increases, prolonged exposure, enhanced stability, or nonspecific antimicrobial effects. By minimizing the verification unit, the influence of these confounding factors can be reduced, allowing the cooperative pathway to emerge as the only plausible explanation. Within the RDE framework, for example: removal of the recognition module tests whether the regulatory module alters the mode of killing rather than merely the quantity; removal of the regulatory module tests whether recognition confers conditional authorization rather than simply locating the target; and removal of the killing module tests whether recognition and decision translate signals into spatiotemporal and intensity thresholds rather than non-selective killing. Only when each minimal binary verification unit is functionally validated can mechanistic significance be established, thereby conferring system-level credibility to the overall ternary collaboration. These minimal binary units correspond exactly to the omission-modulation experimental benchmarks described in section 4.5.

### 6.2 Bacteriophage antibacterial systems versus RDE systems

Phage-based antibacterial systems are generally unsuitable as modular, editable functional slices within RDE systems due to their high degree of endogenous coupling, inherent non-disassemblability, and self-replicating nature. As such, they are not appropriate as tools for the modular design or mechanistic evaluation of RDE-type systems; rather, they are better considered as fully integrated, closed-system references for antibacterial strategy.

In RDE systems, modules must be disassemblable, verifiable, and recombinable. In contrast, bacteriophages, as fully integrated entities, cannot be individually regulated, externally programmed, or quantitatively dose-controlled. It is not feasible to retain only the recognition function of a phage while removing its replication, to preserve its regulatory logic but adjust the killing intensity, or to replace its execution module with antimicrobial peptides or nanomaterials. Such manipulations violate the design principles of RDE systems, precluding application to other modular frameworks and preventing validation via minimal verification units. Consequently, bacteriophage decision-making logic resembles a non-modular hard-wired logic rather than the engineerable modular engineerable logic characteristic of modular RDE systems.

### 6.3 Resistance sensitivity and evolutionary pathways in RDE systems

Compared with traditional continuous-exposure antibacterial strategies, event-driven RDE systems impose fundamentally different pressures on the evolution of drug resistance. In conventional approaches, sustained drug exposure exerts near-constant selective pressure, creating a predictable selective environment for bacteria. Even sub-inhibitory drug concentrations can drive the selection of resistant variants, generating highly reproducible evolutionary trajectories including efflux-pump upregulation, target modification, enhanced membrane-repair mechanisms and reduced metabolic activity (Gullberg et al., 2011). By contrast, the intermittent, heterogeneous, and environmentally contingent exposure characteristic of RDE systems shifts the bacterial survival challenge from “how to endure a drug-laden environment” to “whether the next event will be lethal”. Although the overall selection pressure is not reduced, the RDE system effectively reallocates the adaptive evolutionary burden to the bacteria, altering the form and dynamics of selective pressure rather than its magnitude.

Under certain operational scenarios, ternary RDE-derived modules may produce high-intensity output upon activation, which can amplify even minor bacterial survival advantages and render the system highly sensitive to the natural emergence of drug resistance. This sensitivity underscores the importance of maintaining AMP doses below the threshold required for standalone activity within the RDE framework. The challenge faced by the recognition module is not classical drug resistance, but rather the problem of bacterial “escape.” Acting as a conduit for resistance pathways, the module encounters phenotypic changes, downregulation of virulence factors, masking of metabolic signals, and state transitions that reduce the likelihood of accurate detection and engagement. This is not a direct confrontation; instead, bacteria continuously exploit “overlooked gaps” to evade recognition, highlighting the adaptive strategies that must be anticipated in module design.

Several important evolutionary risks deserve explicit consideration for AND-gate-based RDE architectures. In multi-input AND-logic designs, loss-of-function mutation in any single input pathway will silence killing output, which opens potential bacterial escape routes. Redundant multi-input sensing layers are therefore required to mitigate this vulnerability. Furthermore, classical evolutionary phenomena including bacterial persister cells, antibiotic tolerance, microbial refugia, collateral sensitivity, and cycling therapeutic regimens are highly relevant for evaluating event-driven antibacterial systems (Fisher et al., 2017; Hernando-Amado et al., 2023). Persister subpopulations and refugia can survive intermittent killing pulses even within event-driven schemes. Collateral-sensitivity profiles and cycling-dose strategies may be combined with RDE design to constrain evolutionary paths, but these remain largely untested for modular antimicrobial platforms. We emphasize that reshaping evolutionary selection does not equal elimination of resistance risk.

The decision module serves as the central signal-processing unit of the RDE system, introducing nonlinear thresholds, multi-conditional dependencies, and sequential logic. This complexity may increase the number of evolutionary constraints that bacteria must overcome, but it does not preclude escape through single-pathway loss, phenotypic switching, or persistence (Fisher et al., 2017). In this framework, the adaptive evolutionary burden is fully returned to the bacterium. For this reason, the decision-making pathway must remain unidirectional, well-defined, and low in contextual ambiguity. Any blurring or reversibility of this pathway could transform modules designed to suppress resistance into sources of uncontrolled toxicity and risk (Figure 5).

**Figure 5:**
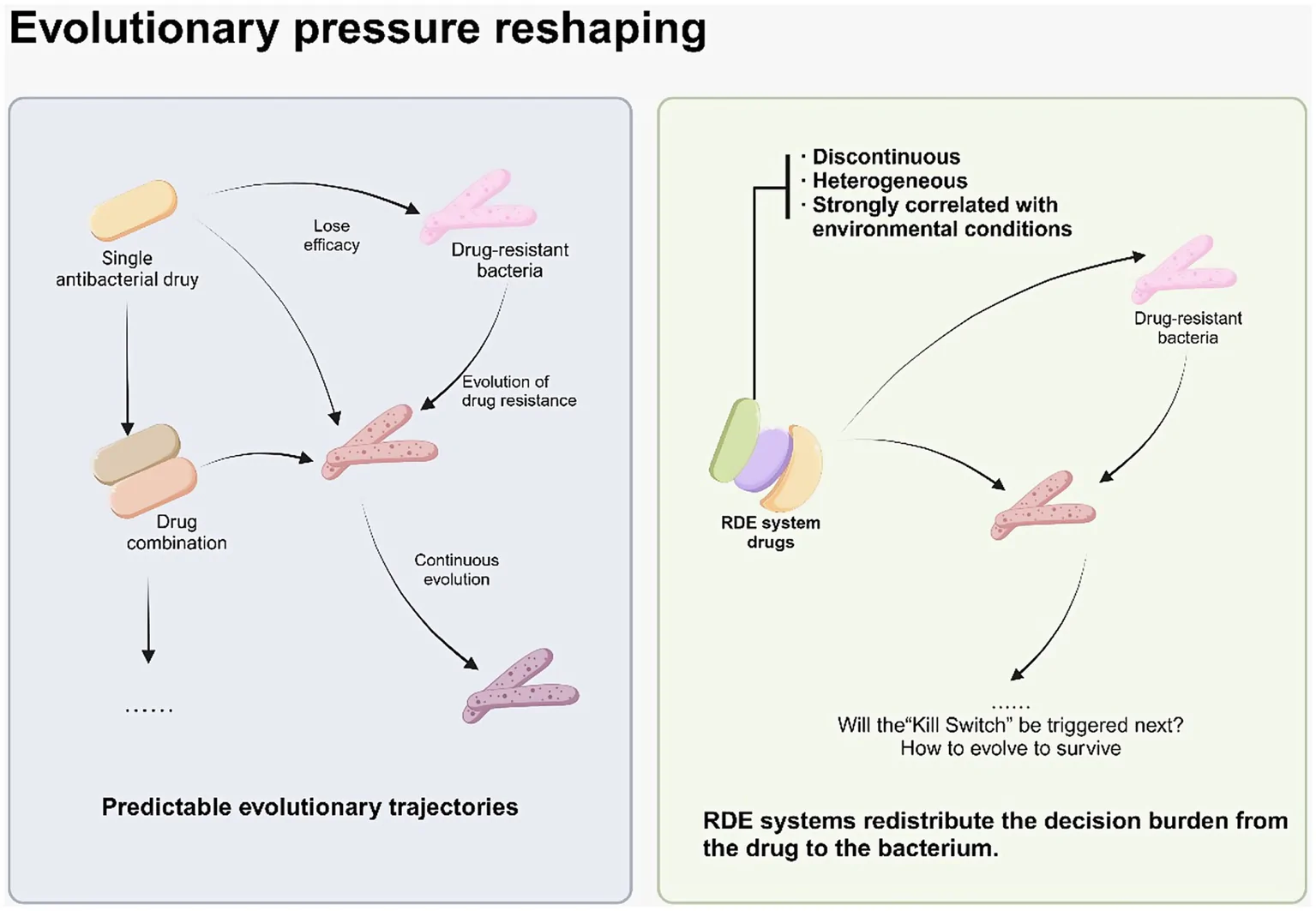
Evolutionary pressure reshaping by authorization-based antibacterial systems. Conventional single agents or drug combinations impose continuous and predictable selective pressure, driving stepwise and directional evolution toward drug resistance. Even combination therapies often converge on similar evolutionary trajectories because killing intensity and timing remain externally fixed. RDE systems replace continuous pressure with discontinuous, context-dependent, and environment-coupled execution, in which antibacterial activity is conditionally authorized rather than constitutively active. As a result, evolutionary outcomes become less predictable, and adaptive burden is redistributed from the drug to the bacterium, which must anticipate when and whether execution will be triggered. This represents a conceptual hypothesis illustrating how authorization-based antibacterial systems may reshape evolutionary pressure. It remains a testable hypothesis rather than broadly validated experimental conclusion. RDE systems do not guarantee suppression of stable resistance fixation.

From this perspective, the evolution of resistance is driven not solely by lethality, but by continuous exposure and predictability. It raises the question of whether the future competitiveness of antimicrobial systems should shift from the pursuit of increasingly potent killing modules to the development of more “intelligent” decision-making modules, thereby controlling the evolutionary trajectory of bacterial resistance at its source. This reshaping of evolutionary pressure represents a testable hypothesis; long-term *in vitro* evolution and in-vivo infection experiments remain necessary to confirm whether stable resistance fixation can be suppressed.

## 7 Challenges and limitations

RDE systems should not be interpreted as ready-to-use therapeutic regimens for clinical translation. On the contrary, increasing the number of functional modules often amplifies system-level uncertainty. When multiple synergistic interactions lack well-defined functional boundaries, or when the decision-making pathway is no longer unidirectional, the intensity of killing can undergo nonlinear transitions. Modules introduced to enhance efficacy may instead elicit unpredictable tissue toxicity or immune activation. Importantly, these risks do not stem from any single molecule or material per se, but from the systemic consequences of uncontrolled coordination within a collaborative network.

While functional slices expand the freedom of design, they inevitably introduce new systemic risks, particularly with respect to toxicity amplification, nonspecific adsorption, and immune activation. Multi-level recognition and regulatory mechanisms can, in theory, enhance precision; however, in practical systems, they may also generate transient “peaks” of instantaneous killing intensity through local enrichment or signal cascade effects. These peaks need not be persistent to significantly amplify local toxicity. Such effects are often not captured in safety assessments of individual materials or modules and are more accurately considered emergent “side effects” of system-level synergy. This highlights that the value of a regulatory module lies not in indefinitely amplifying responses, but in being finely tuned to “back off” at critical junctures. High-affinity recognition units, multivalent ligand architectures, and high-surface-energy nanostructures often carry an inherent risk of nonspecific adsorption. In complex humoral environments, such off-target interactions can not only dilute recognition accuracy, but also trigger misactivation in non-target tissues. Consequently, relying on a single recognition signal is insufficient to sustain a high-reliability guidance system over extended periods. Multi-condition gating and sequential recognition strategies are therefore not merely advantageous enhancements, but may represent an inevitable direction for system development.

Beyond material-related toxicity, immunogenicity and non-specific adsorption, system-level failure originating from recognition-decision modules deserves systematic consideration. Recognition modules may suffer off-target interference caused by protein corona in biological fluids, leading to erroneous target recognition. Noise within complex in-vivo microenvironments can satisfy decision-module threshold conditions accidentally and trigger false-positive authorization. Unintended activation of execution effectors may then produce off-target killing of commensal microbiota or host tissue injury. Multi-condition AND-gate logic, carefully calibrated activation thresholds, and multi-layer state-validation could mitigate such risks, yet these strategies introduce additional complexity to system fabrication (Liu et al., 2024). Multi-component assembly also brings practical hurdles including batch-to-batch reproducibility, colloidal stability in biological media and manufacturing scalability (Moore et al., 2015; Kelly et al., 2026; Desai, 2012).

It should also be emphasized that common sensing cues exploited in current antibacterial platforms (pH shift, lipase activity, reactive oxygen species) represent infection-associated microenvironmental markers rather than strictly pathogen-specific biomarkers. Consequently, RDE architectures can reduce but cannot guarantee complete protection of commensal microbiota; off-target activation against commensals remains a realistic risk when host microenvironment signals are perturbed in non-infectious contexts. Achieving true pathogen-specific activation will require incorporation of highly pathogen-unique molecular signatures.

RDE-type architectures offer unique potential advantages particularly for chronic biofilm-associated infections where preservation of commensal microbiota is desired. Nevertheless, event-dependent authorization brings inherent activation latency; such systems may not be well-suited for acute life-threatening sepsis requiring immediate unconditional bactericidal action.

Finally, it is necessary to confront a frequently overlooked but unavoidable challenge: partial recognition or regulatory modules-including exogenous nanomaterials, phage-derived structures, or surface modifications with inherent immunogenicity-may themselves elicit unexpected immune activation. Such responses can either pose safety hazards or, under specific circumstances, be harnessed as part of the antimicrobial response. The critical question, therefore, is not whether immune activation can be entirely avoided, but whether it can be maintained within a predictable and controllable range. Moreover, the complexity inherent to modular assembly renders the system highly sensitive to conformational changes, component ratios, and even microenvironmental fluctuations. This sensitivity not only amplifies manufacturing challenges but also complicates quality control. For instance, the *in vivo* stability, nonspecific adsorption, and potential immunogenicity of aptamers continue to constrain their direct clinical use as recognition modules. Beyond these molecular considerations, multi-component systems remain immature with respect to cost management, regulatory pathways, and long-term safety evaluation.

For these reasons, the RDE system should not be interpreted merely as a “more complex combination strategy”, but rather as a conceptual framework and methodological approach. Its purpose is not to provide a standardized treatment immediately, but to offer a new way of organizing antimicrobial action-shifting from a constant-pressure paradigm toward event-driven processes through functional decoupling, information transfer, and decision-making empowerment. Within this framework, antibacterial design moves beyond indiscriminate killing. It begins to account for the potential evolution of resistance, the management of systemic toxicity, and adaptability to complex microenvironments. In other words, the RDE framework provides a conceptual paradigm and set of testable design hypotheses for next-generation, evolution-aware antibacterial strategies.

In this sense, the RDE system does not mark the culmination of antibacterial complexity, but rather represents the starting point for an engineering-, information-, and evolution-informed approach to antimicrobial design.
